# Glucagon-Like Peptide-1 Receptor Agonists in Hip and Knee Arthroplasty: A Comprehensive Systematic Review and Meta-Analysis of Perioperative and Postoperative Outcomes

**DOI:** 10.1016/j.artd.2026.102043

**Published:** 2026-05-16

**Authors:** Benjamin Wajda, Darren Van Essen, Sabrina Martini, Golpira Elmi Assadzadeh, Rajrishi Sharma, Jason Werle

**Affiliations:** a Department of Orthopaedic Surgery, University of Calgary, Calgary, Alberta, Canada; b Department of Orthopaedic Surgery, University of Manitoba, Winnipeg, Manitoba, Canada; c Department of Internal Medicine, University of Calgary, Calgary, Alberta, Canada

**Keywords:** Glucagon-like peptide-1 receptor agonists, Knee arthroplasty, Hip arthroplasty, Complications, Outcomes, Infection

## Abstract

**Background:**

Obesity drives demand for total hip arthroplasty (THA) and total knee arthroplasty (TKA) and increases the risks of infection, wound complications, and revision. Glucagon-like peptide-1 receptor agonists (GLP-1RAs) are increasingly used for metabolic optimization, but their perioperative safety and effect on arthroplasty outcomes remain unclear. This study evaluated associations between perioperative GLP-1RA use and postoperative outcomes following THA and TKA.

**Methods:**

A Preferred Reporting Items for Systematic Reviews and Meta-Analyses-compliant systematic review identified studies of adults undergoing primary or revision THA/TKA with perioperative GLP-1RA exposure (0–12 months preoperatively or ≤30 days postoperatively). PubMed, MEDLINE, and EMBASE were searched through August 20, 2025. Data on demographics, surgical and medical complications, and resource use were collected at 90 days and 1–2 years. Fixed- and random-effects meta-analyses pooled odds ratios (ORs) with 95% confidence intervals (CIs).

**Results:**

Thirteen retrospective cohorts including 1,408,609 patients (39,614 THA; 1,373,771 TKA) were analyzed. GLP-1RA use was associated with reduced 90-day periprosthetic joint infection (OR: 0.77; 95% CI: 0.66–0.89), lower 90-day all-cause revision (OR: 0.88; 95% CI: 0.82–0.95), and decreased 90-day readmission (OR: 0.79; 95% CI: 0.70–0.89). A 1-year increase in periprosthetic fracture risk was observed (OR: 1.49; 95% CI: 1.08–2.07), mainly in TKA. No associations were seen for venous thromboembolism, pulmonary embolism, renal failure, pneumonia, cardiac arrest, or aspiration.

**Conclusions:**

Perioperative GLP-1RA use in THA and TKA improves early infection, revision, and readmission outcomes without increasing short-term medical or aspiration risk. The observed 1-year fracture risk warrants further study. Prospective trials are needed to define optimal timing and patient selection.

## Introduction and background

Obesity is a major driver of the rising demand for total hip arthroplasty (THA) and total knee arthroplasty (TKA) [1] and markedly increases the risk of knee osteoarthritis and progression to end-stage disease requiring surgery [[1], [2], [3]]. By 2029, most primary TKAs (69%) and THAs (55%) are projected to be performed in obese patients [4]. Obesity is associated with higher rates of perioperative complications—including deep infection, poor wound healing, and revision—prompting many centers to adopt body mass index cutoffs of ≥40 kg/m^2^ despite concerns regarding access to care [[5], [6], [7], [8]]. As a result, interest has grown in glucagon-like peptide-1 receptor agonists (GLP-1RAs) for preoperative weight loss and metabolic optimization in this high-risk population.

GLP-1RAs, including semaglutide (Ozempic, Wegovy), exenatide (Byetta), and liraglutide (Victoza), were initially developed for glycemic control in type 2 diabetes but are now widely used for their potent weight-loss effects [[9], [10], [11]]. In the United States, spending on GLP-1 medications increased more than 500% from 2018 to 2023 and is expected to continue rising [12]. These agents enhance satiety, suppress glucagon secretion, and delay gastric emptying, promoting glycemic control and weight reduction [13,14]. GLP-1RAs have important musculoskeletal effects that may be relevant in arthroplasty populations. They reduce absolute lean mass—accounting for approximately 25% of total weight loss—though emerging evidence suggests improvements in muscle quality and preserved short-term strength [15]. These agents increase bone turnover, with a relatively greater effect on resorption markers, while demonstrating modest improvements in bone mineral density at the lumbar spine, femoral neck, and total hip in patients with type 2 diabetes. Notably, these effects appear to vary by metabolic status, with greater bone loss observed in nondiabetic populations, particularly at weight-bearing sites [16,17].

Given the increasing prevalence of obesity among patients undergoing total hip and knee arthroplasty, GLP-1RAs may offer a promising avenue for preoperative weight loss, metabolic optimization, and improved clinical outcomes in the surgical hip and knee arthroplasty population. Although their use has been extensively studied in the context of weight and diabetes management, their application in arthroplasty remains poorly. This systematic review and meta-analysis synthesizes current evidence on perioperative GLP-1RA use and its association with postoperative outcomes, including venous thromboembolism (VTE), periprosthetic fracture (PPFx), prosthetic joint infection (PJI), revision, and length of stay (LOS).

## Material and methods

### Literature search

A systematic review and meta-analysis was performed in accordance with the Preferred Reporting Items for Systematic Reviews and Meta-Analyses (PRISMA) 2020 guidelines for systematic reviews and meta-analysis *(PROSPERO ID: CRD420251130439*) [18,19]. A comprehensive literature search was conducted across the PubMed, MEDLINE, and EMBASE databases through August 20, 2025, to identify peer-reviewed, original research articles evaluating the use of GLP-1RAs in preoperative and perioperative patients undergoing hip and knee arthroplasty (Supplementary Table 1).

### Inclusion criteria

**Figure 1:**
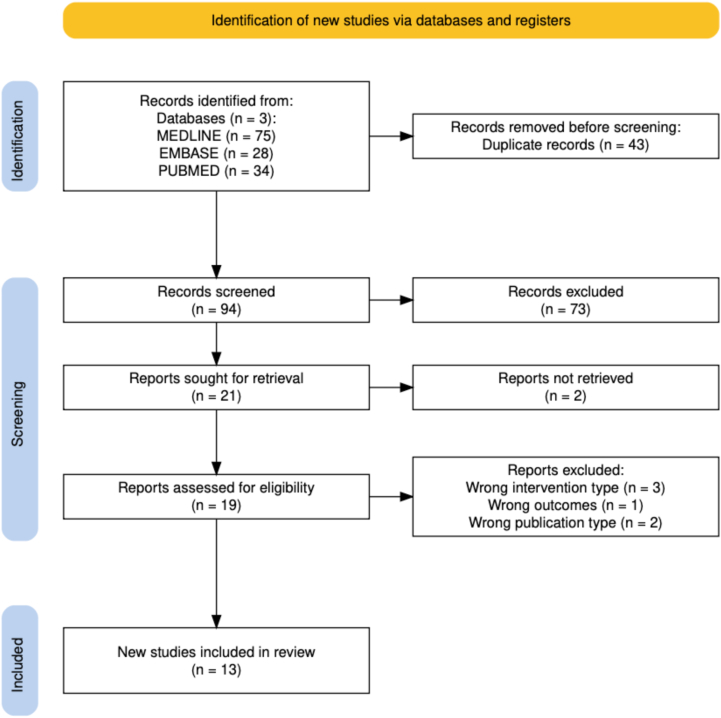
PRISMA flow diagram illustrating the study selection process for meta-analysis and systematic review of GLP-RA in hip and knee arthroplasty patients.

Studies were included if they examined adults (≥18 years) undergoing elective primary or revision THA or TKA who received a GLP-1RA—such as semaglutide (Ozempic and Wegovy), liraglutide (Victoza), exenatide (Bydureon), or dulaglutide (Trulicity)—during the preoperative (0–12 months) or perioperative period (≤30 days postoperatively). Eligible studies needed to utilize a GLP-1RA for either weight-loss, diabetes, or both and were required to report at least one relevant clinical or surgical outcome, including postoperative complications (eg, infection, wound complications, PPFx, VTE, aspiration, pneumonia), revision, hospital readmission, and LOS. Randomized trials and observational studies (cohort, case-control, and retrospective designs) published in peer-reviewed journals were included. Case reports, abstracts, protocols, editorials, reviews, nonhuman research, non-English articles, and studies without a comparison group were excluded. Studies not involving GLP-1RA use, nonsurgical populations, or arthroplasty outside the hip or knee were also excluded. Two independent reviewers (B.W. and D.V.E.) screened all studies using Covidence (Veritas Health Innovation, Melbourne, Australia), with a third reviewer (S.M.) resolving disagreements. The search yielded 137 records; 94 unique articles remained after duplicate removal, 19 underwent full-text review, and 13 met the inclusion criteria for final synthesis [[20], [21], [22], [23], [24], [25], [26], [27], [28], [29], [30], [31], [32], [33]] (Fig. 1).

### Quality assessment

**Figure 2:**
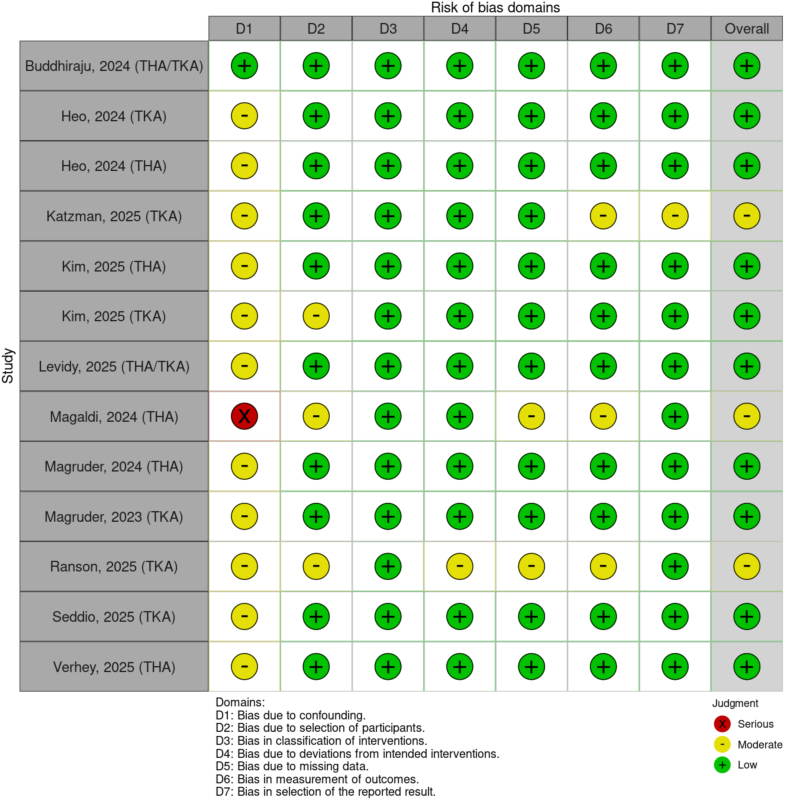
Risk of Bias in Non-Randomized Studies of Interventions (ROBINS-I).

The quality of included studies was assessed using the Newcastle–Ottawa Scale, a standardized tool developed explicitly for evaluating the methodological rigor of observational cohort and case-control studies [34]. Risk of bias was evaluated separately using the ROBINS-I tool (Risk Of Bias In Non-Randomized Studies of Interventions), a Cochrane framework designed to assess bias in non-randomized intervention studies [35] (Fig. 2, Supplementary Table 2). All included studies were published in the United States between 2023 and 2025. Average Newcastle–Ottawa Scale was determined to be 6.9 and 10 studies reported low bias and 3 reported moderate bias (Fig. 2 and Supplementary Table 3).

### Data extraction

Data extraction was performed independently by 2 reviewers using a standardized form. Variables included study characteristics, patient demographics, GLP-1RA exposure, and all reported postoperative outcomes. Surgical outcomes were collected at 90 days, 1 year, and 2 years, including all-cause revision, PPFx, PJI, wound dehiscence, hematoma, and superficial surgical site infection (SSI). Medical outcomes, consistently reported at 90 days, included deep vein thrombosis (DVT), pulmonary embolism (PE), acute renal failure, cerebrovascular accidents, hypoglycemic events, pneumonia, mortality, aspiration, and cardiac arrest. Healthcare resource utilization measures were also extracted, including 90-day readmission, emergency department utilization, and LOS. For each outcome, the effect measure (odds ratio [OR] or mean difference [MD]) and 95% confidence interval (CI) were recorded, along with hip- and knee-specific subgroup estimates when available. When multiple postoperative intervals were reported, all relevant time points were captured (Supplementary Table 3).

### Data analysis

Data synthesis and statistical analysis were performed using R (Version 4.4). Meta-analyses used fixed- and random-effects models to estimate pooled effect sizes with 95% CIs. For binary outcomes, event counts and sample sizes were analyzed using the metabin function, while continuous outcomes (eg, LOS) were assessed using metacont. Results are presented as forest plots, with study weights primarily determined by sample size. Pooled analyses followed PRISMA guidelines [19]. Heterogeneity was assessed using the chi-square test and inconsistency index (I^2^), with significance defined as *P* < .10 or I^2^ > 50%. I^2^ values of 0–30% were considered low, 31–70% moderate, and 71–100% substantial. Statistical significance was set at *P* < .05. Hip and knee arthroplasty studies were analyzed as separate subgroups, with an overall pooled estimate also reported. Sensitivity analyses assessed the influence of the largest study (Ranson et al.). Publication bias was evaluated using funnel plots and Egger’s test, with trim-and-fill applied when asymmetry was detected to estimate adjusted pooled effects.

## Results

### Patient characteristics

The pooled cohort comprised 1,373,771 TKA patients and 34,838 THA patients. In the TKA group, the weighted mean age was 65.8 years, with 63.0% female, 86.1% obese, 36.5% diabetic, and 10.9% smokers. In the THA group, the weighted mean age was 59.4 years, with 55.3% female, 79.4% obese, 62.0% diabetic, and 34.1% smokers. The TKA estimates were heavily influenced by the large cohort reported by Ranson et al. [30] (Table 1). Of the 13 included studies, 10 showed no meaningful differences in comorbidities between groups after propensity matching. Magaldi et al. reported higher preoperative hemaglobin A1c, lower Medicaid coverage, and greater prevalence of diabetes, insulin use, and metformin use in the GLP-1RA group [23]. Kim et al. reported lower rates of alcohol abuse and smoking among GLP-1RA users [24], while Ranson et al. observed higher rates of diabetes, congestive heart failure, peripheral vascular disease, and obesity in the GLP-1RA group [30].

**Table 1:** Summary of patient cohort characteristics of the enrolled studies.

Author, year	Country	Procedure	Sample size	Age	% female	BMI	% obesity	% DM	Tobacco use
Buddhiraju, 2024	United States	THA	2088	63.4	51.5	-	71.7	68.8	-
		TKA	4190	64.2	60.8	-	69.7	68.5	-
Heo, 2024	United States	TKA	4776	61.1	57.0	-	-	100	12.5
Heo, 2025	United States	THA	4060	60.5	42.3	-	-	100	17.9
Katzman, 2025	United States	TKA	9515	64.0	66.3	36.0	-	40.9	6.2
Kim, 2025	United States	THA	3855	62.1	52.9	>/ = 40.0	100	52.3	-
Kim, 2025	United States	TKA	5950	62.2	66.9	>/ = 40.0	100	52.8	48.2
Levidy, 2025	United States	THA	4488	-	-	-	-	100	-
		TKA	9400	-	-	-	-	100	-
Magaldi, 2024	United States	THA	192	67.1	42.2	34.9	-	-	-
Magruder, 2023	United States	TKA	31,575	-	61.6	-	86.4	100	42.3
Magruder, 2024	United States	THA	9465	-	47.8	-	85.3	100	45.8
Ranson, 2025	United States	TKA	1,300,656	65.9	63.0	44.2	-	33.7	9.8
Seddio, 2025	United States	TKA	7709	65.2	67.2	-	82.9	100	43.3
Verhey, 2025	United States	THA	10,690	57.0	68.8%	-	68.3	0	29.9

BMI, body mass index; DM, diabetes mellitus; -, not reported.

### Surgical outcomes

**Figure 3:**
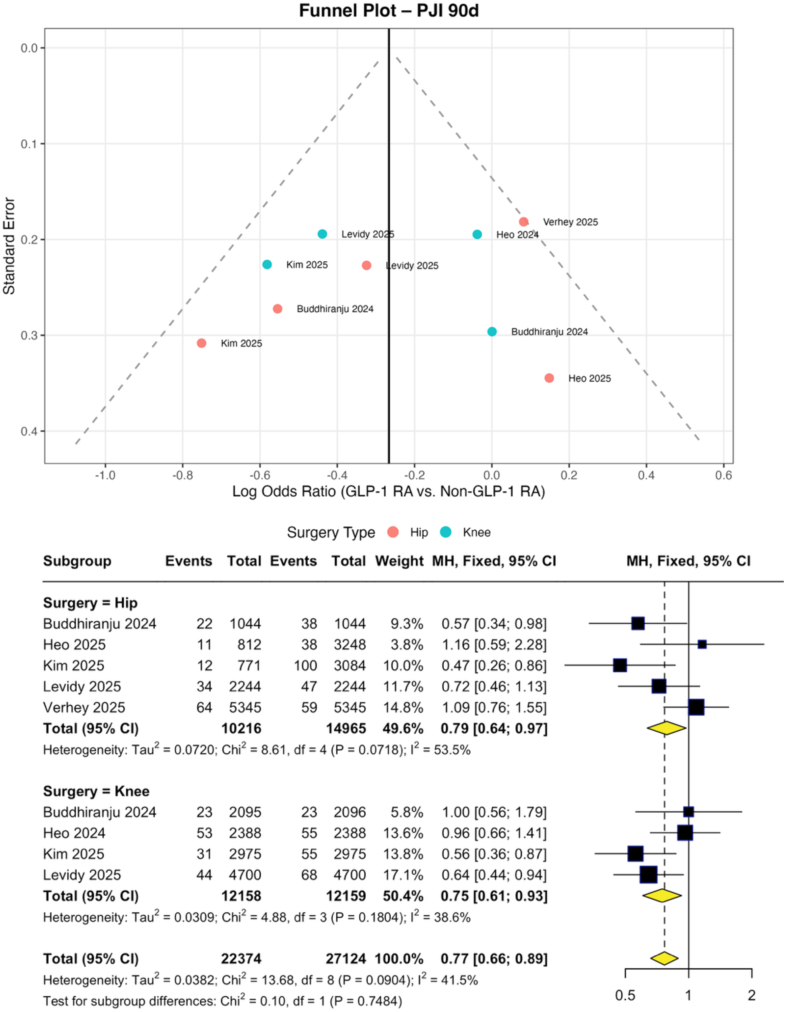
Funnel and forest plots of pooled and subgroup meta-analysis of periprosthetic infection at 90 days.

**Figure 4:**
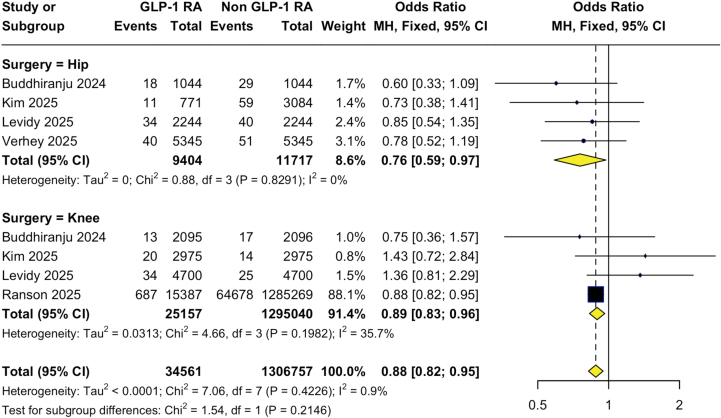
Forest plots of pooled and subgroup meta-analysis of all-cause revision at 90 days.

**Supplementary Figure 1:**
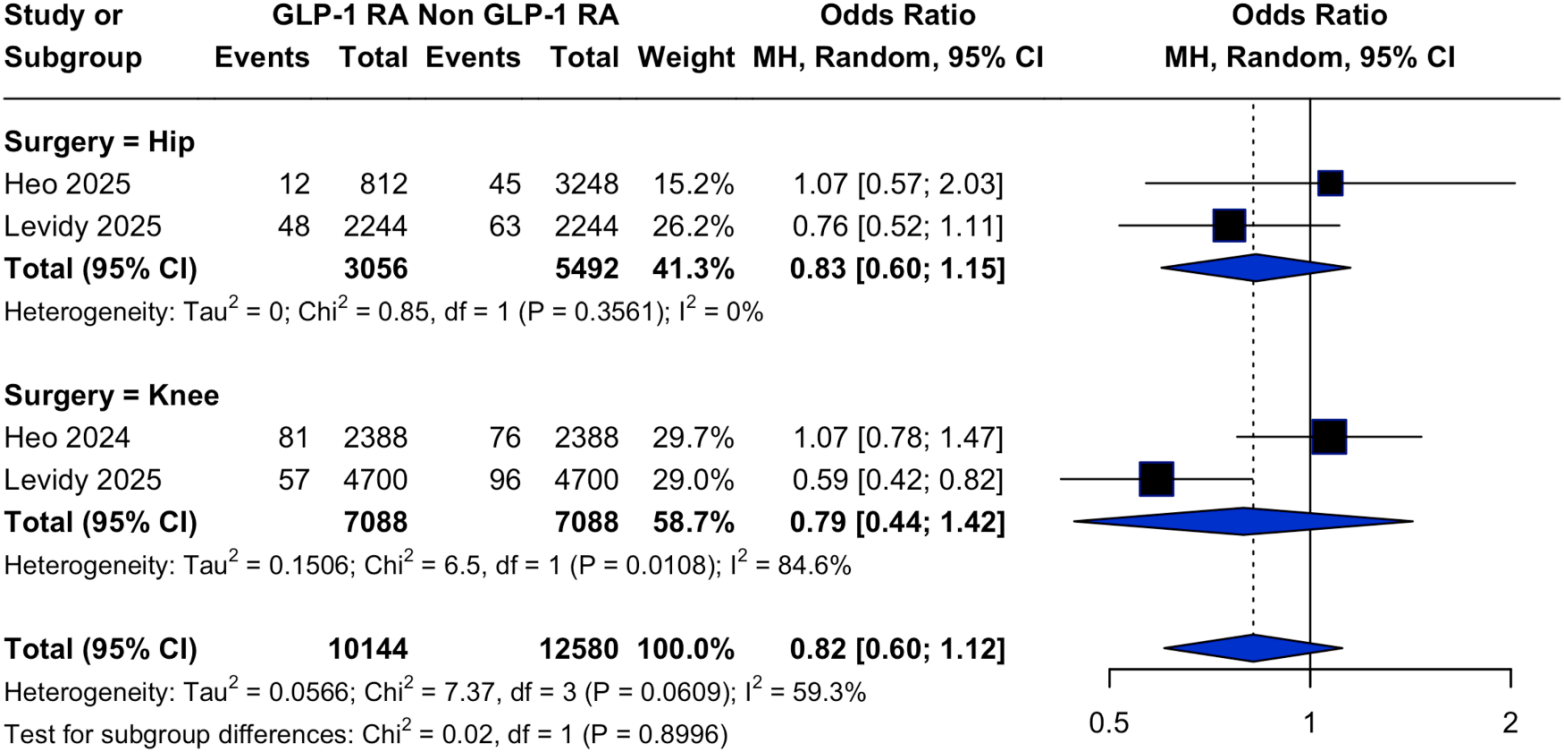
Pooled and subgroup meta-analysis of periprosthetic joint infection at 1 year.

**Supplementary Figure 2:**
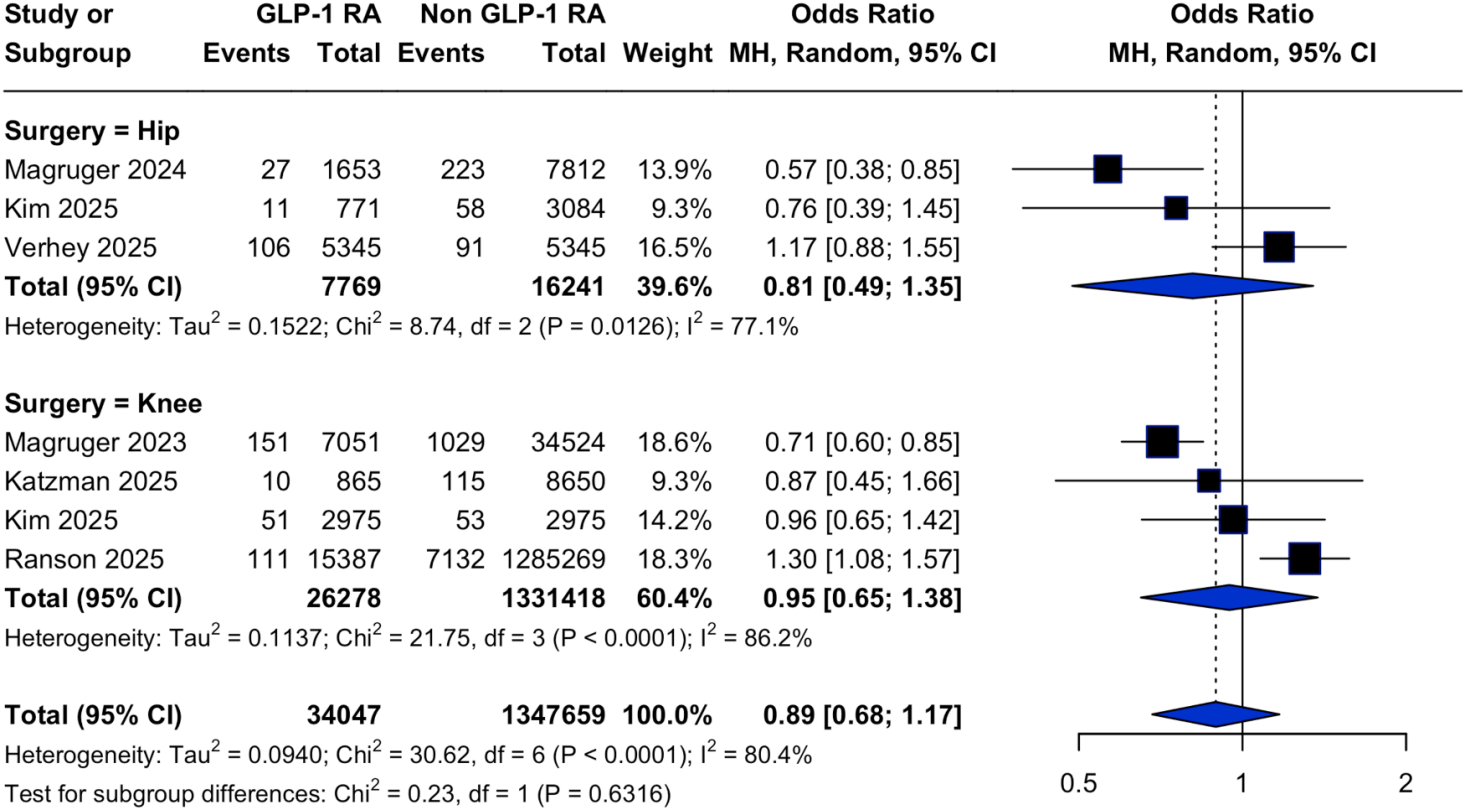
Pooled and subgroup meta-analysis of periprosthetic joint infection at 2 years.

**Supplementary Figure 3:**
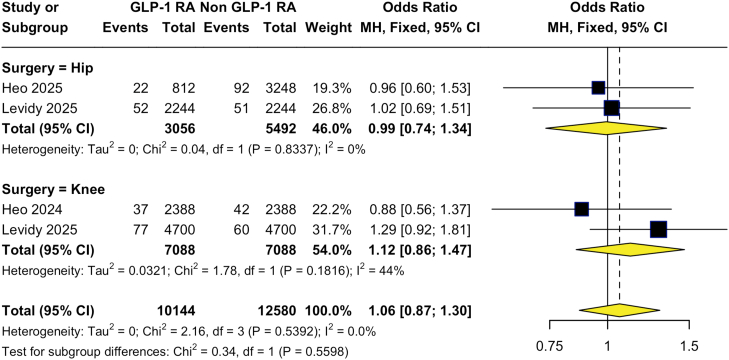
Pooled and subgroup meta-analysis of all-cause revision at 1 year.

**Supplementary Figure 4:**
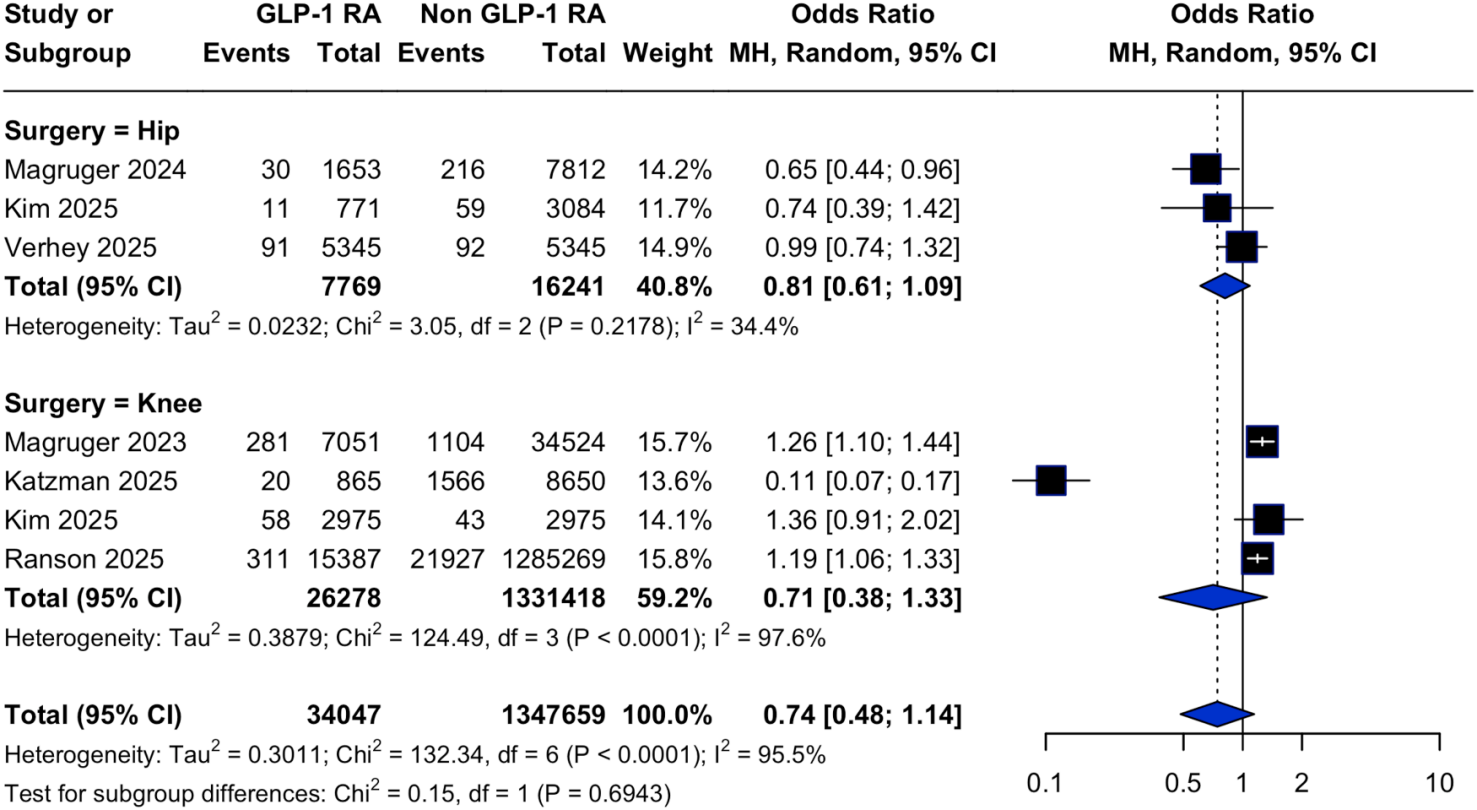
Pooled and subgroup meta-analysis of all-cause revision at 2 years.

**Supplementary Figure 5:**
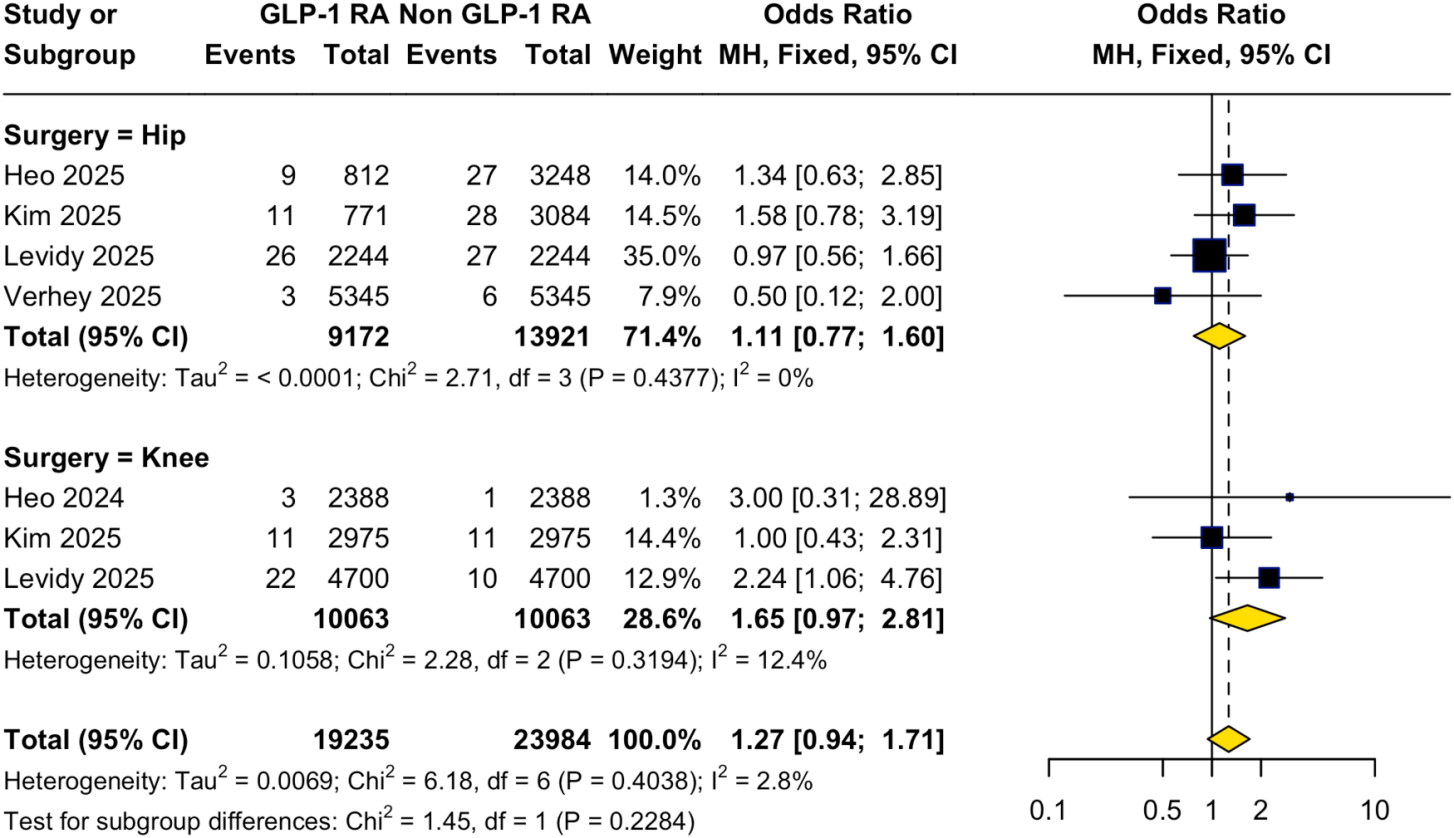
Pooled and subgroup meta-analysis of periprosthetic fracture at 90 days.

**Supplementary Figure 6:**
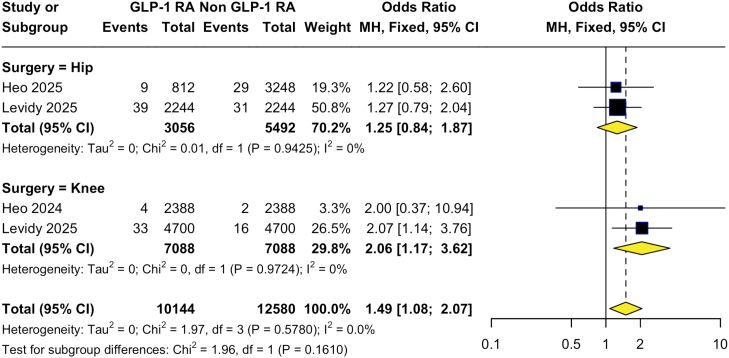
Pooled and subgroup meta-analysis of periprosthetic fracture at 1 year.

**Supplementary Figure 7:**
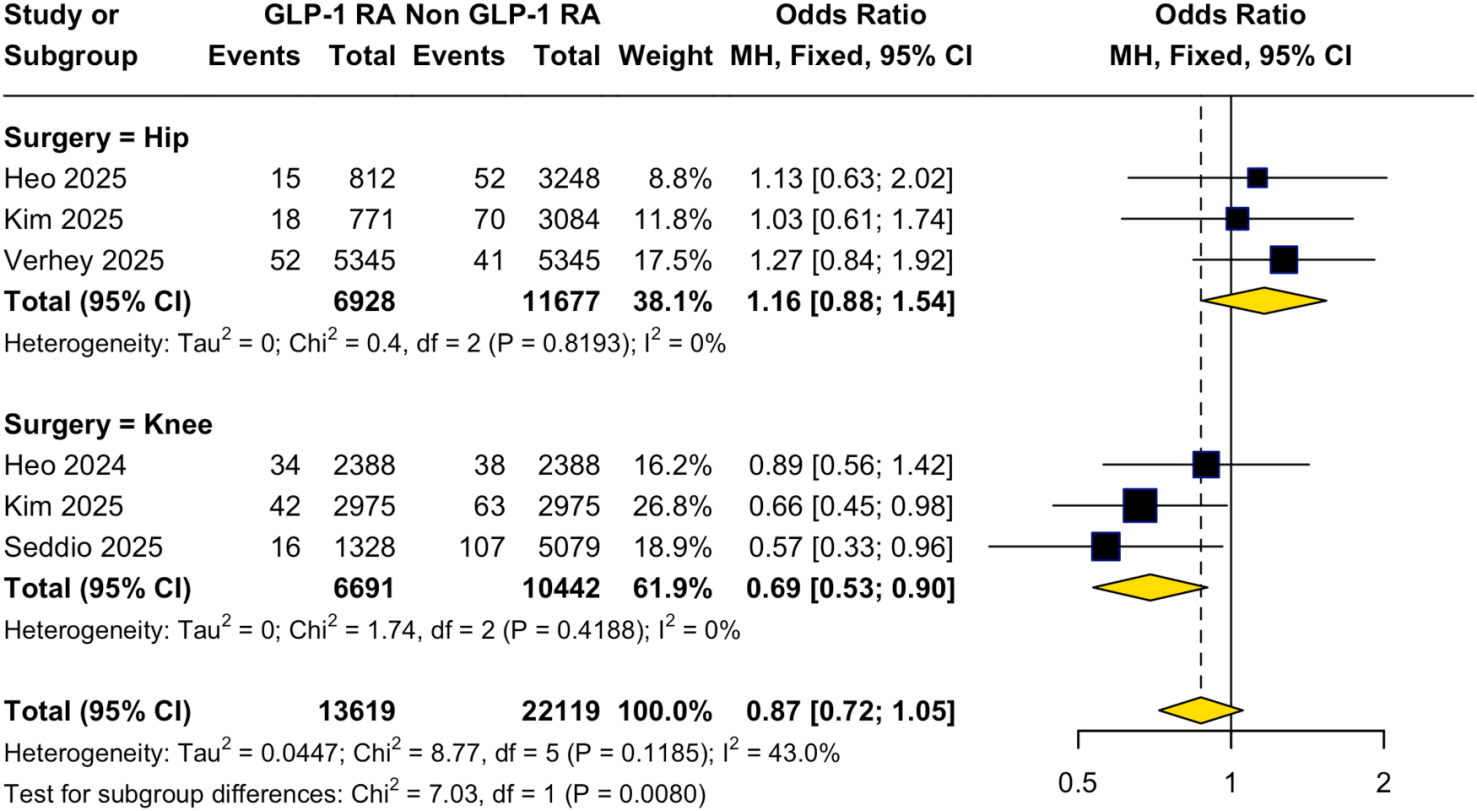
Pooled and subgroup meta-analysis of wound dehiscence at 90 days.

Across included studies, GLP-1RA use demonstrated time- and outcome-specific associations after hip and knee arthroplasty. GLP-1RA use was associated with a reduced risk of early PJI at 90 days (pooled OR: 0.77; 95% CI: 0.66–0.89), with consistent effects in both THA (OR: 0.79; 95% CI: 0.64–0.97) and TKA (OR: 0.75; 95% CI: 0.61–0.93); however, no associations were observed at 1 year (OR: 0.82; 95% CI: 0.60–1.12) or 2 years (OR: 0.89; 95% CI: 0.68–1.17). In contrast, a time-dependent relationship was observed for PPFx, with a nonsignificant increase at 90 days (OR: 1.27; 95% CI: 0.94–1.71) and a significant association at 1 year (OR: 1.49; 95% CI: 1.08–2.07), driven primarily by TKA (OR: 2.06; 95% CI: 1.17–3.62), but not at 2 years (OR: 0.85; 95% CI: 0.38–1.86). GLP-1RA use was also associated with lower early revision risk at 90 days (OR: 0.88; 95% CI: 0.82–0.95), with similar effects in THA (OR: 0.76; 95% CI: 0.59–0.97) and TKA (OR: 0.89; 95% CI: 0.83–0.96), but not at 1 year (OR: 1.06; 95% CI: 0.87–1.30) or 2 years (OR: 0.74; 95% CI: 0.48–1.14). Wound outcomes showed procedure-specific effects: GLP-1RA use was not associated with wound dehiscence after THA (OR: 1.16; 95% CI: 0.88–1.54) but was associated with a reduced risk after TKA (OR: 0.69; 95% CI: 0.53–0.90; *P* = .008). No significant associations were observed for SSI in either THA (OR: 0.93; 95% CI: 0.71–1.23) or TKA (OR: 0.72; 95% CI: 0.44–1.19), and the pooled effect was also nonsignificant (OR: 0.83; 95% CI: 0.63–1.10). Results are summarized in Figure 3, Figure 4 and Table 2, Table 3, Supplementary Table 4, Supplementary Figure 1, Supplementary Figure 2, Supplementary Figure 3, Supplementary Figure 4, Supplementary Figure 5, Supplementary Figure 6, Supplementary Figure 7.

**Table 2:** Summary of 90-day surgical outcomes of enrolled studies.

Author, year	Procedure	Group (N)	Superficial SSI	Wound dehiscence	Hematoma	All-cause revision	PJI	PPFx
Buddiranju, 2024	THA	Control (1044)	10 (1.0%)	-	-	29 (2.8%)	38 (4.2%)	-
		GLP-1RA (1044)	10 (1.0%)	-	-	18 (1.7%)	22 (2.5%)	-
	TKA	Control (2095)	11 (0.5%)	-	-	17 (0.8%)	23 (1.1%)	-
		GLP-1RA (2095)	13 (0.6%)	-	-	13 (0.6%)	23 (1.1%)	-
Heo, 2024	TKA	Control (2388)	108 (4.5%)	38 (1.4%)	-	-	55 (2.3%)	1 (0.1%)
		GLP-1RA (2388)	112 (4.7%)	34 (1.4%)	-	-	53 (2.2%)	3 (0.1%)
Heo, 2025	THA	Control (3248)	170 (5.2%)	51 (1.6%)	-	-	38 (1.2%)	27 (0.8%)
		GLP-1RA (812)	32 (3.9%)	15 (1.8%)	-	-	11 (1.3%)	9 (1.1%)
Kim, 2025	THA	Control (3084)	-	70 (2.3%)	39 (1.3%)	59 (1.9%)	100 (3.2%)	28 (0.9%)
		GLP-1RA (771)	-	18 (2.3%)	0 (0%)	11 (1.4%)	12 (1.6%)	11 (1.4%)
Kim, 2025	TKA	Control (2975)	-	63 (2.1%)	14 (0.5%)	14 (0.5%)	55 (1.8%)	11 (0.4%)
		GLP-1RA (2975)	-	42 (1.4%)	12 (0.4%)	20 (0.7%)	31 (1.0%)	11 (0.4%)
Levidy, 2025	THA	Control (2244)	27 (1.2%)	-	-	40 (1.8%)	47 (2.1%)	27 (1.2%)
		GLP-1RA (2244)	26 (1.2%)	-	-	34 (1.5%)	34 (1.5%)	26 (1.2%)
	TKA	Control (4700)	-	-	-	25 (0.5%)	68 (1.4%)	10 (0.2%)
		GLP-1RA (4700)	-	-	-	34 (0.7%)	44 (0.9%)	22 (0.5%)
Magruder, 2024	THA	Control (7812)	106 (1.4%)	-	-	-	-	-
		GLP-1RA (1653)	17 (1.0%)	-	-	-	-	-
Magruder, 2023	TKA	Control (34524)	-	-	-	-	-	-
		GLP-1RA (7051)	-	-	-	-	-	-
Ranson, 2025	TKA	Control (1285269)	18378 (1.4%)	-	-	-	-	-
		GLP-1RA (15387)	192 (1.2%)	-	-	-	-	-
Seddio, 2025	TKA	Control (5079)	259 (5.1%)	107 (2.1%)	56 (1.1%)	-	-	-
		GLP-1RA (1328)	18 (1.4%)	16 (1.2%)	11 (0.8%)	-	-	-
Verhey, 2025	THA	Control (5345)	22 (0.4%)	41 (0.8%)	23 (0.4%)	51 (1.0%)	59 (1.1%)	6 (0.1%)
		GLP-1RA (5345)	34 (0.6%)	52 (1.0%)	16 (0.3%)	40 (0.7%)	64 (1.2%)	3 (0.1%)

-, not reported.

**Table 3:** Summary of meta-analysis results for surgical outcomes.

Outcome	Procedure	GLP-1RA (n)	Non-GLP-1RA (n)	Pooled OR	95% CI (5th)	95% CI (95th)	*P* value	I^2^	Heterogeneity
90-day all-cause revision	THA	9404	11,717	0.76	0.59	0.97	.8291	0	None
	TKA	25,157	1,295,040	0.89	0.83	0.96	.1982	35.7	Low
	Combined	34,561	1,306,757	0.88	0.88	0.96	.4226	0.9	Low
1-year all-cause revision	THA	3056	5492	0.99	0.74	1.34	.8337	0	None
	TKA	7088	7088	1.12	0.86	1.47	.1816	44	Moderate
	Combined	10,144	12,580	1.06	0.87	1.30	.5392	0	None
2-year all-cause revision	THA	7769	16,241	0.81	0.61	1.09	.2178	34.4	Moderate
	TKA	26,278	1,331,418	0.71	0.38	1.33	<.0001	97.6	Substantial
	Combined	34,047	1,347,659	0.74	0.48	1.14	<.0001	95.5	Substantial
90-day PJI	THA	10,216	14,965	0.79	0.64	0.97	.0718	53.5	Moderate
	TKA	12,158	12,159	0.75	0.61	0.93	.1804	38.6	Moderate
	Combined	22,374	27,124	0.77	0.66	0.89	.0904	41.5	Moderate
1-year PJI	THA	3056	5492	0.83	0.60	1.15	.3561	0	None
	TKA	7088	7088	0.79	0.44	1.42	.0108	84.6	Substantial
	Combined	10,144	12,580	0.82	0.60	1.12	.0609	59.3	Moderate
2-year PJI	THA	7769	16,241	0.81	0.49	1.35	.0126	77	Substantial
	TKA	26,278	1,331,418	0.95	0.65	1.38	<.0001	86	Substantial
	Combined	34,047	1,347,659	0.89	0.68	1.17	<.001	80.4	Substantial
90-day PPFx	THA	9172	13,921	1.11	0.77	1.60	.4377	0	None
	TKA	10,063	10,063	1.65	0.97	2.81	.3194	12.4	Low
	Combined	19,235	23,984	1.27	0.94	1.71	.4038	2.8	Low
1-year PPFx	THA	3056	5492	1.25	0.84	1.87	.9425	0	None
	TKA	7088	7088	2.06	1.17	3.62	.9724	0	None
	Combined	10,144	12,580	1.49	1.08	2.07	.5780	0	None
2-year PPFx	THA	7769	16,241	0.68	0.09	5.37	.0014	84.8	Substantial
	TKA	25,413	1,312,768	0.78	0.30	2.02	.0453	67.7	Moderate
	Combined	33,182	1,329,009	0.78	0.38	1.86	.0020	73.6	Substantial
Superficial SSI	THA	11,098	19,693	0.93	0.71	1.23	.2442	26.6	Low
	TKA	21,198	12,943,821	0.72	0.44	1.19	<.0001	89	Substantial
	Combined	32,296	1,314,524	0.83	0.63	1.10	<.0001	75.8	Substantial
Wound dehiscence	THA	6928	11,677	1.16	0.88	1.54	.8193	0	None
	TKA	6691	10,442	0.69	0.53	0.90	.4188	0	None
	Combined	13,619	22,119	0.87	0.72	1.05	.1185	43	Moderate

### Medical outcomes

**Supplementary Figure 8:**
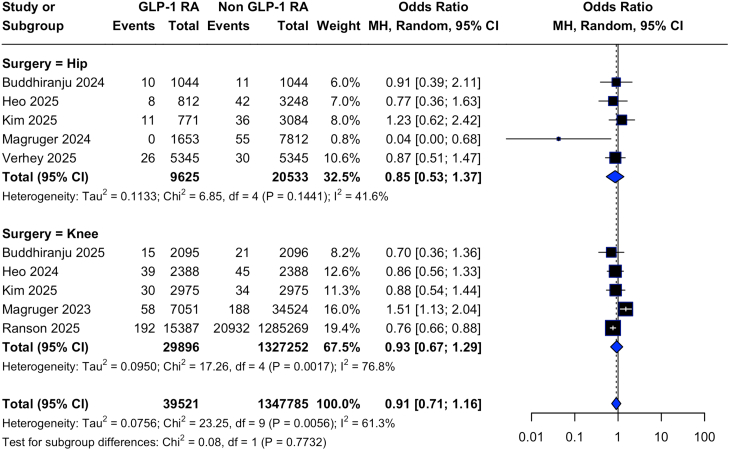
Pooled and subgroup meta-analysis of deep-vein thrombosis at 90 days.

**Supplementary Figure 9:**
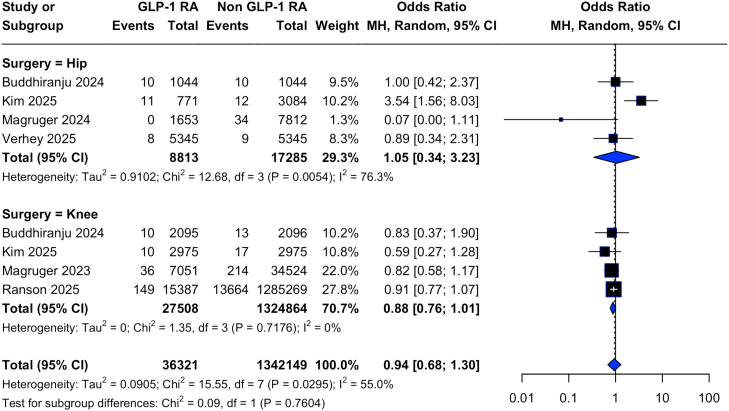
Pooled and subgroup meta-analysis of pulmonary embolism at 90 days.

At 90 days, medical complications showed no meaningful association with GLP-1RA use across evaluated postoperative events. There was no difference in DVT risk after hip (OR: 0.85; 95% CI: 0.53–1.37) or knee arthroplasty (OR: 0.93; 95% CI: 0.67–1.29), with a nonsignificant pooled estimate (OR: 0.91; 95% CI: 0.71–1.16). PE risk was likewise unchanged for hip (OR: 1.05; 95% CI: 0.34–3.23) and knee procedures (OR: 0.88; 95% CI: 0.76–1.01), with no overall effect (OR: 0.94; 95% CI: 0.68–1.30) Table 4, Table 5 and Supplementary Figure 8, Supplementary Figure 9.

**Table 4:** Summary of 90-day venous thromboembolism outcomes of the enrolled studies.

Author, year	Procedure	Group (N)	DVT	PE
Buddiranju, 2024	THA	Control (1044)	11 (1.0%)	10 (1.0%)
		GLP-1RA (1044)	10 (1.0%)	10 (1.0%)
	TKA	Control (2095)	21 (1.0%)	13 (0.6%)
		GLP-1RA (2095)	15 (0.7%)	11 (0.5%)
Heo, 2024	TKA	Control (2388)	45 (1.9%)	-
		GLP-1RA (2388)	39 (1.6%)	-
Heo, 2025	THA	Control (3248)	43 (1.3%)	-
		GLP-1RA (812)	8 (1.0%)	-
Kim, 2025	THA	Control (3084)	36 (1.2%)	12 (0.4%)
		GLP-1RA (771)	11 (1.4%)	11 (1.4%)
Kim, 2025	TKA	Control (2975)	34 (1.1%)	17 (0.6%)
		GLP-1RA (2975)	30 (1.0%)	10 (0.3%)
Magruder, 2024	THA	Control (7812)	55 (0.7%)	34 (0.4%)
		GLP-1RA (1653)	0	0
Magruder, 2023	TKA	Control (34524)	188 (0.5%)	214 (0.6%)
		GLP-1RA (7051)	58 (0.8%)	36 (0.5%)
Ranson, 2025	TKA	Control (1285269)	20932 (1.6%)	13664 (1.1%)
		GLP-1RA (15387)	192 (1.2%)	149 (1.0%)
Seddio, 2025	TKA	Control (5079)	-	-
		GLP-1RA (1328)	-	-
Verhey, 2025	THA	Control (5345)	30 (0.6%)	9 (0.2%)
		GLP-1RA (5345)	26 (0.5%)	8 (0.15%)

**Table 5:** Summary of meta-analysis results for medical outcomes and resource outcomes.

Outcome	Procedure	GLP-1RA (n)	Non-GLP-1RA (n)	Pooled OR	95% CI (5th)	95% CI (95th)	*P* value	I^2^	Heterogeneity
90-day readmission	THA	9625	20,533	0.8	0.42	1.52	.1546	40	Low
	TKA	30,761	1,335,901	0.78	0.66	0.92	<.0001	82.8	Substantial
	Combined	40,386	1,356,434	0.79	0.70	0.89	<.0001	72.1	Moderate
DVT	THA	9625	20,533	0.85	0.53	1.37	.1441	41.6	Low
	TKA	29,896	1,327,252	0.93	0.67	1.29	.0017	76.8	Moderate
	Combined	39,521	1,347,785	0.91	0.71	1.16	.0056	61.3	Moderate
Pulmonary embolism	THA	8813	17,285	1.05	0.34	3.23	.0054	76.3	Moderate
	TKA	27,508	1,324,864	0.88	0.76	1.01	.7176	0	None
	Combined	36,321	1,342,149	0.94	0.68	1.30	.0295	55	Low
Length of stay imputed SD	THA	24,900	11,022	−0.3	−1.7	1.1	<.0001	96.9	Substantial
	TKA	10,891	46,149	−0.3	−0.6	−0.0	.0037	82.1	Substantial
	Combined	13,381	57,171	−0.3	−0.7	0.0	<.0001	93.4	Substantial

## Resource outcomes

**Figure 5:**
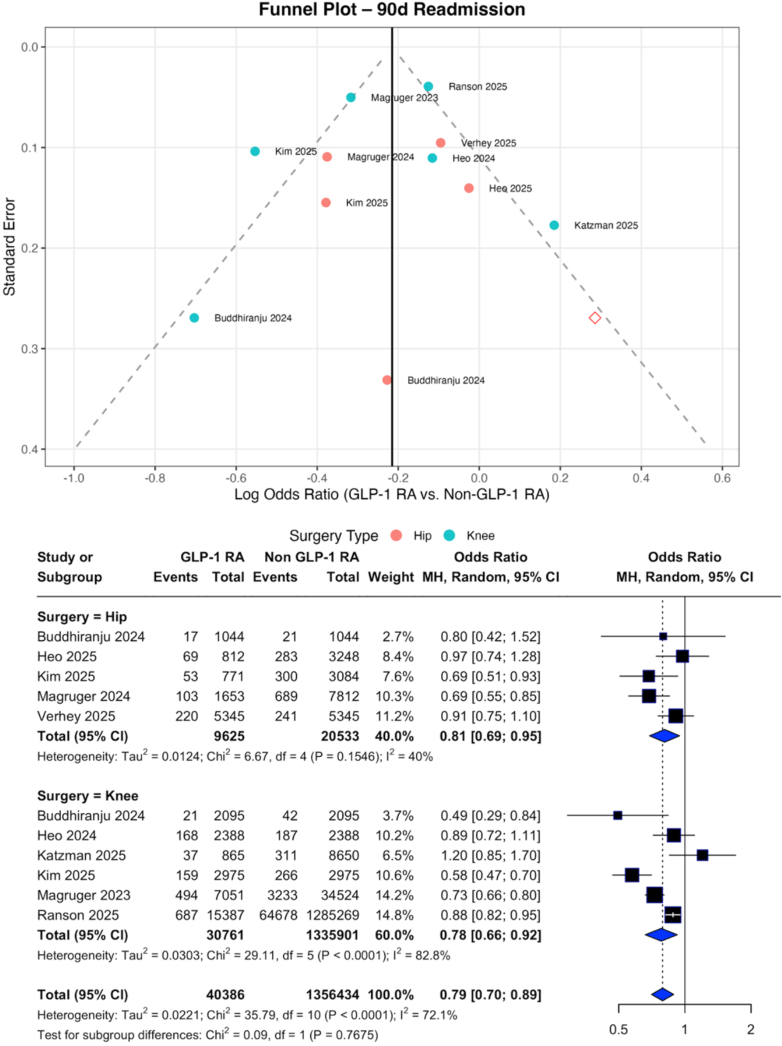
Funnel and forest plots of pooled and subgroup meta-analysis of 90-day readmission.

GLP-1RA therapy was associated with a meaningful reduction in 90-day readmission following both hip (OR: 0.81; 95% CI: 0.69–0.95) and knee arthroplasty (OR: 0.78; 95% CI: 0.66–0.92), yielding a significant pooled benefit (OR: 0.79; 95% CI: 0.70–0.89). No significant overall reduction in hospital LOS was observed (MD –0.3 days; 95% CI: –0.7 to 0.0), although a modest decrease was noted for knee arthroplasty alone (MD –0.3 days; 95% CI: –0.6 to −0.0) (Fig. 5, Supplementary Table 5).

### Sensitivity analysis

To evaluate robustness, multiple sensitivity analyses were performed. A leave-one-out approach sequentially removed each study, including the large Ranson et al. cohort; its inclusion or omission did not meaningfully alter the magnitude, direction, or significance of any outcome. Subgroup analyses by surgical site (hip vs knee) and follow-up interval (90 days, 1 year, 2 years) showed consistent results. Heterogeneity was assessed using I^2^, and estimates were compared between fixed- and random-effects models to confirm stability. Across all analyses, the main findings—reduced 90-day PJI risk, reduced revision and readmission risk, and increased 1-year PPFx risk—remained unchanged. Additional analyses focused on the Ranson cohort confirmed no change in statistical significance for superficial SSI, 90-day readmission, 2-year all-cause revision, 2-year PJI, or 2-year PPFx. Its primary effect was improved precision for superficial SSI, with narrower CIs and reduced heterogeneity, particularly in TKA. Finally, excluding TriNetX cohorts with imputed event counts (assigned a value of 10) produced materially unchanged estimates, indicating minimal impact from imputation.

## Discussion

The use of GLP-RAs in the perioperative setting for THA and TKA is increasingly relevant as obesity and type 2 diabetes rise [36]. In this systematic review and meta-analysis of 13 studies including 1,408,609 arthroplasty patients, GLP-1RA use was associated with heterogeneous but clinically meaningful postoperative outcomes. The most consistent benefit of GLP-1RA use was observed in early postoperative outcomes, including reduced 90-day PJI (OR: 0.77; 95% CI: 0.66–0.89), early all-cause revision (OR: 0.88; 95% CI: 0.82–0.95), and 90-day readmission (OR: 0.79; 95% CI: 0.70–0.89). Wound complications showed procedure-specific patterns: GLP-1RA use lowered dehiscence in TKA (OR: 0.69; 95% CI: 0.53–0.90) but not in THA, while other wound and superficial SSI outcomes showed no associations. Mechanistic data support these findings, as GLP-1RAs reduce inflammation via nuclear factor kappa-light-chain-enhancer of activated B-cells (NF-κB) inhibition, suppress tumor necrosis factor (TNF-α) and interleukin-6 (IL-6), and promote anti-inflammatory macrophage phenotypes [37,38]. Improved glycemic control, reduced adiposity, and weight-loss–associated metabolic stability likely contribute to these early benefits [[38], [39], [40], [41]]. Importantly, these results should be interpreted with caution. Most patients were likely prescribed GLP-1RAs for type 2 diabetes rather than weight loss (TKA: 36.5% diabetic, 86.1% obese; THA: 62.0% diabetic, 79.4% obese), so the observed associations may reflect underlying metabolic status or comorbidity rather than a direct drug effect, limiting causal inference. Longer-term outcomes (1- and 2-year PJI and revision) showed no significant differences, suggesting predominantly short-term protection, though point estimates remained directionally favorable, warranting confirmation in prospective studies.

Our findings suggest that GLP-1RA exposure may confer a time-dependent increase in PPFx risk. At 90 days, we observed a nonsignificant trend toward higher PPF rates among GLP-1RA users (OR: 1.65; 95% CI: 0.97–2.81), which progressed to a statistically significant elevation by 1 year (OR: 2.06; 95% CI: 1.17–3.62), driven primarily by TKA, with no clear effect in THA. This temporal pattern aligns with prior arthroplasty literature, including a recent study showing higher PPF incidence in GLP-1RA users after TKA at both 3 months (0.47% vs 0.21%) and 1 year (0.70% vs 0.34%) [26]. Together, these data support a growing signal that GLP-1RA may influence bone quality or mechanical loading, resulting in increased susceptibility to PPF over time, rather than acute perioperative impairment.

Mechanistically, GLP-1RA–induced rapid weight loss leads to mechanical unloading of cortical bone, increased resorption, and a transient high-turnover state that may impair periprosthetic bone strength during remodeling, consistent with evidence that GLP-1–mediated metabolic shifts can disrupt skeletal homeostasis [42,43]. Concurrent lean mass loss may alter biomechanics and loading across the implant, while microarchitectural deterioration (eg, increased cortical porosity) and nutritional deficits further compromise bone quality and healing [44,45]. The TKA-specific effect likely reflects the greater reliance on cortical bone at the distal femur and proximal tibia and altered joint mechanics. The apparent reduction at 2 years likely reflects data sparsity or survivorship bias rather than true reversal [30,46]. While meta-analyses in broader diabetic populations suggest neutral or reduced fracture risk with long-term GLP-1RA use, the discrepancy in arthroplasty patients likely reflects baseline bone differences and perioperative factors [47]. Overall, these findings indicate a transient, mechanistically plausible increase in PPF risk after TKA.

Venous thromboembolism events (DVT and PE) were not significantly influenced by GLP-1RA use, reinforcing the overall systemic safety of these agents in the perioperative period. Length of stay reductions were minimal and limited to knee arthroplasty, highlighting modest resource benefits, though they may lead to cost-effectiveness.

Seddio et al. [31] showed that <1 month of semaglutide reduced minor complications, whereas ≥2–3 months were needed to reduce major events such as sepsis, VTE, and PJI, with no added benefit beyond 12 months. Xie et al. demonstrated reduced long-term revision when GLP-1RAs were initiated postoperatively with modest weight loss [48], supporting metabolic optimization both before and after surgery. Cost analyses of preoperative semaglutide use in THA and TKA were inconclusive, with no significant differences in same-day or 90-day costs, though trends favored lower 90-day costs—particularly in TKA [28,29]. One study evaluated patient-reported outcomes using the Hip Osteoarthritis Outcome Score for Joint Replacement and found no differences in patient-reported outcomes between GLP-1 agonist users and matched controls from preoperative assessment to 12 months, although scores were slightly lower in GLP-1 users (favorable) at 6 and 12 months [27].

Overall, these findings suggest that GLP-1RA therapy is generally safe and may provide meaningful early postoperative benefits in infection, revision, and readmission risk, while highlighting an increase in fracture risk at 1 year. These conclusions should be interpreted in light of several limitations. All included studies were retrospective cohorts, subject to selection bias, unmeasured confounding, and methodological heterogeneity. Sample sizes varied widely (192 to >1.3 million), with several studies using overlapping administrative databases raising the possibility of duplicate patients. Outcomes were dependent on coding accuracy, and the lack of granular clinical data precluded assessment of perioperative glycemic control, weight change, or thresholds of weight loss. The study period (2005–2024) also spans evolving surgical practices and GLP-1RA prescribing patterns. A key limitation is heterogeneity in the indication for GLP-1RA use. Seven studies included only diabetic patients, one study included only patients using GLP-1RAs for weight loss (which showed no increased risk of postoperative complications), and 5 included mixed populations (average 52.3% diabetic). As GLP-1RAs are now more recently adopted for weight loss, most patients were likely treated for type 2 diabetes. While outcomes were similar between diabetic-only and mixed cohorts, the inability to distinguish indication at the patient level limits interpretation. We could not determine whether observed benefits were due to GLP-1RA therapy itself, improved glycemic control, weight loss, or underlying metabolic differences. Moreover, with only aggregate data available, patient-level analyses to evaluate indication-specific effects were not possible, limiting causal inference. Variation in diabetes and obesity status and moderate heterogeneity across outcomes further reduce certainty. Socioeconomic factors were not captured in these datasets, and given the high cost and variable insurance coverage of GLP-1RAs, observed benefits may partly reflect differences in access to care rather than direct pharmacologic effects. Furthermore, 5 studies reported higher-than-expected tobacco use rates, likely reflecting differences in coding (eg, “any tobacco use” vs “active smoking”), which may introduce further variability in risk adjustment. Prospective cohort trials—especially in nondiabetic populations and elective orthopaedic settings—are needed to more rigorously define the safety and efficacy of perioperative GLP-1RA use.

## Conclusions

This meta-analysis of 13 retrospective cohort studies involving 1,408,609 THA and TKA patients demonstrates that perioperative GLP-1RA use is associated with meaningful early postoperative benefits. Users had lower 90-day risks of PJI, revision, and readmission, with reduced wound dehiscence observed in TKA. GLP-1RA use was not associated with increased VTE, including DVT or PE. A transient increase in PPFx risk at 1 year, particularly in TKA, was observed, highlighting a potential delayed skeletal effect. While benefits were largely early, these findings support a potential role for GLP-1RAs in perioperative optimization and underscore the need for prospective studies to clarify timing, duration, and fracture risk mitigation strategies in arthroplasty.

## CRediT authorship contribution statement

**Benjamin Wajda:** Writing – review & editing, Writing – original draft, Visualization, Validation, Supervision, Methodology, Investigation, Data curation, Conceptualization. **Darren Van Essen:** Writing – review & editing, Writing – original draft, Methodology, Investigation, Data curation. **Sabrina Martini:** Writing – review & editing, Writing – original draft, Methodology, Investigation, Conceptualization. **Golpira Elmi Assadzadeh:** Writing – review & editing, Formal analysis. **Rajrishi Sharma:** Writing – review & editing, Supervision, Conceptualization. **Jason Werle:** Writing – review & editing, Supervision, Conceptualization.

## Conflict of interest

J. Werle is on the speakers bureau/paid presentations for Zimmer Biomet Inc; is a paid employee for Institute for Improved Health Outcomes; receives other financial or material support from Zimmer Biomet, Stryker, DePuy, and Smith & Nephew; and is a board member/committee appointments for Canadian Arthroplasty Society. R. Sharma is on the speakers bureau/paid presentations for DePuy Synthes and Stryker and is a paid consultant for DePuy Synthes and Stryker; all other authors declare no potential conflicts of interest.

For full disclosure statements refer to https://doi.org/10.1016/j.artd.2026.102043.

## Figures and Tables

**Supplementary Table 1:** Database search strategy.

Database	MEDLINE (Ovid), EMBASE (Ovid), PubMed
Date of search	August 20, 2025
Search combination	Population AND Intervention
Line	Search Terms
1	exp Glucagon-Like Peptide-1 Receptor Agonists/
2	Glucagon-Like Peptide-1 Receptor Agonist∗.tw,kf.
3	semaglutide∗.tw,kf.
4	GLP-1.tw,kf.
5	ozempic.tw,kf.
6	wegovy.tw,kf.
7	rybelsus.tw,kf.
8	1 or 2 or 3 or 4 or 5 or 6 or 7
9	exp Arthroplasty, Replacement, Hip/
10	exp Arthroplasty, Replacement, Knee/
11	((knee∗ or hip∗) adj3 (arthroplast∗ or replac∗ or procedur∗ or surg∗)).tw,kf.
12	9 or 10 or 11
13	8 and 12

**Supplementary Table 2:** Newcastle-Ottawa Scale (NOS) summary of included studies.

Study	Selection (0-4)	Comparability (0-3)	Outcome (0-3)	Total NOS (10)
Buddhiraju, 2024 (THA/TKA)	★★★★	★★	★★★	9
Heo, 2025 (THA)	★★★	★★	★★	7
Heo, 2024 (TKA)	★★★	★★	★★	7
Katzman, 2025 (TKA)	★★★	★	★★	6
Kim, 2024 (THA)	★★★	★★	★★	7
Kim, 2025 (TKA)	★★★	★★	★★	7
Levidy, 2025 (THA/TKA)	★★★	★★	★★	7
Magaldi, 2024 (TKA)	★★	★	★★	5
Magruder, 2024 (THA)	★★★★	★★	★★	8
Magruder, 2023 (TKA)	★★★★	★★	★★	8
Ranson, 2025 (TKA)	★★	★	★★	5
Seddio, 2025 (TKA)	★★★	★★	★★	7
Verhey, 2025 (THA)	★★★	★★	★★	7

NOS, Newcastle-Ottawa Scale (0-10).

**Supplementary Table 3:** Summary of study characteristics of included studies (n = 13) with Newcastle-Ottawa Scale (NOS).

First author, y	Country	Design	Data source	Data period	Follow-up duration	Propensity matching	Total NOS
Buddhiraju, 2024	United States	Retrospective cohort	TriNetx Global Collaborative Network	May 2005-December 2023	90 d	1:1	9
Heo, 2024	United States	Retrospective cohort	IBM MarketScan Databases	January 2016-December 2021	1 y	1:1	7
Heo, 2025	United States	Retrospective cohort	IBM MarketScan Databases	January 2016-December 2021	1 y	1:4	7
Katzman, 2025	United States	Retrospective cohort	Institutional Health Records	January 2012-November 2023	5 y	1:10	6
Kim, 2025	United States	Retrospective cohort	PearlDiver Database	January 2010-October 2022	2 y	1:1	7
Kim, 2025	United States	Retrospective cohort	PearlDiver Database	January 2010-December 2022	2 y	1:4	7
Levidy, 2025	United States	Retrospective cohort	TriNetx Global Collaborative Network	2005-2024	1 y	1:1	7
Magaldi, 2024	United States	Retrospective cohort	Institutional Health Records	June 2016-December 2022	1 y	1:2	5
Magruder, 2023	United States	Retrospective cohort	PearlDiver Database	January 2010-March 2021	2 y	1:5	8
Magruder, 2024	United States	Retrospective cohort	PearlDiver Database	January 2010-October 2021	2 y	1:5	8
Ranson, 2025	United States	Retrospective cohort	PearlDiver Database (M165Ortho)	2010-2022	1 y	NA	5
Seddio, 2025	United States	Retrospective cohort	PearlDiver Database (M165Ortho)	January 2010-July 2022	2 y	1:4	7
Verhey, 2025	United States	Retrospective cohort	PearlDiver Database	January 2010-January 2022	2 y	1:1	7

**Supplementary Table 4:** Summary of 1- and 2-year primary surgical outcomes reported in each of the enrolled.

Author, y	Procedure	Group (N)	1 y:				2 y:			
			All-cause revision	Aseptic revision	PJI	PPFx	All-cause revision	Aseptic revision	PJI	PPFx
Buddhiraju, 2024	THA	Control (1044)	-	-	-	-	-	-	-	-
		GLP-1RA (1044)	-	-	-	-	-	-	-	-
	TKA	Control (2095)	-	-	-	-	-	-	-	-
		GLP-1RA (2095)	-	-	-	-	-	-	-	-
Heo, 2024	TKA	Control (2388)	42 (1.8%)	27 (1.1%)	76 (3.2%)	2 (0.2%)	-	-	-	-
		GLP-1RA (2388)	37 (1.5%)	24 (1.0%)	81 (3.4%)	4 (0.1%)	-	-	-	-
Heo, 2025	THA	Control (3248)	92 (2.8%)	87 (2.7%)	46 (1.4%)	28 (0.9%)	-	-	-	-
		GLP-1RA (812)	22 (2.7%)	20 (2.5%)	12 (1.5%)	9 (1.1%)	-	-	-	-
Katzman, 2025	TKA	Control (8650)	-	-	-	-	224 (2.6%)	109 (1.3%)	-	-
		GLP-1RA (865)	-	-	-	-	20 (2.3%)	10 (1.2%)	-	-
Kim, 2025	THA	Control (3084)	-	-	-	-	59 (3.1%)	-	58 (3.1%)	15 (0.8%)
		GLP-1RA (771)	-	-	-	-	11 (2.3%)	-	11 (2.3%)	11 (2.3%)
Kim, 2025	TKA	Control (2975)	-	-	-	-	43 (2.4%)	-	53 (3.0%)	11 (0.6%)
		GLP-1RA (2975)	-	-	-	-	58 (3.3%)	-	51 (2.9%)	11 (0.6%)
Levidy, 2025	THA	Control (2244)	51 (2.3%)	-	63 (2.8%)	31 (1.4%)	-	-	-	-
		GLP-1RA (2244)	52 (2.3%)	-	48 (2.1%)	39 (1.7%)	-	-	-	-
	TKA	Control (4700)	60 (1.3%)	-	96 (2.0%)	16 (0.3%)	-	-	-	-
		GLP-1RA (4700)	77 (1.6%)	-	57 (1.2%)	33 (0.7%)	-	-	-	-
Magruder, 2024	THA	Control (7812)	-	-	-	-	216 (2.8%)	-	223 (2.9%)	39 (0.5%)
		GLP-1RA (1653)	-	-	-	-	30 (1.8%)	-	27 (1.6%)	0 (0.0%)
Magruder, 2023	TKA	Control (34524)	-	-	-	-	1566 (4.5%)	-	1029 (3.0%)	40 (0.1%)
		GLP-1RA (7051)	-	-	-	-	281 (4.0%)	-	151 (2.1%)	0 (0.0%)
Ranson, 2025	TKA	Control (1285269)	-	-	-	-	21927 (0.2%)	-	7132 (0.6%)	3609 (0.3%)
		GLP-1RA (15387)	-	-	-	-	311 (0.2%)	-	111 (0.5%)	48 (0.3%)
Verhey, 2025	THA	Control (5345)	-	-	-	-	92 (1.7%)	-	91 (1.7%)	6 (0.1%)
		GLP-1RA (5345)	-	-	-	-	91 (1.7%)	-	106 (2.0%)	4 (0.07%)

**Supplementary Table 5:** Summary of resource utilization reported outcomes in each of the enrolled studies.

Author, y	Procedure	Group (N)	Readmission	LOS (mean ± SD)	Extended LOS >3d	ED utilization	Average cost (90d)	Average same-day cost
Buddhiraju, 2024	THA	Control (1044)	21 (2.0%)	-	-	69 (6.6%)	-	-
		GLP-1RA (1044)	17 (1.6%)	-	-	62 (5.9%)	-	-
	TKA	Control (2095)	42 (2.0%)	-	-	161 (7.7%)	-	-
		GLP-1RA (2095)	23 (1.1%)	-	-	151 (7.2%)	-	-
Heo, 2024	TKA	Control (2388)	187 (7.8%)	-	746 (31.2%)	-	-	-
		GLP-1RA (2388)	168 (7.0%)	-	607 (25.4%)	-	-	-
Heo, 2025	THA	Control (3248)	284 (8.7%)	-	926 (28.5%)	-	-	-
		GLP-1RA (812)	69 (8.5%)	-	198 (24.4%)	-	-	-
Katzman, 2025	TKA	Control (8650)	309 (4.3%)	2.5 ± 4.8	-	350 (4.0%)	-	-
		GLP-1RA (865)	37 (4.3%)	2.1 ± 3.3	-	51 (5.9%)	-	-
Kim, 2025	THA	Control (3084)	300 (9.7%)	3.1 ± 4.0	-	-	-	-
		GLP-1RA (771)	53 (6.9%)	2.2 ± 1.4	-	-	-	-
Kim, 2025	TKA	Control (2975)	266 (8.9%)	2.9 ± 1.8	-	-	-	-
		GLP-1RA (2975)	159 (5.3%)	2.7 ± 2.3	-	-	-	-
Magaldi, 2024	THA	Control (66)	-	2.4 ± 0.8	-	-	-	-
		GLP-1RA (126)	-	2.6 ± 0.9	-	-	-	-
Magruder, 2024	THA	Control (7812)	103 (6.2%)	2.9 ± 2.3	-	-	$14,681.71	$10,046.30
		GLP-1RA (1653)	689 (8.8%)	2.70 ± 1.8	-	-	$13,219.92	$9174.72
Magruder, 2023	TKA	Control (34524)	3233 (9.4%)	3.1 ± 2.7	-	-	$16,798.46	$11,484.50
		GLP-1RA (7051)	491 (7.0%)	2.70 ± 1.9	-	-	$15,291.66	$10,671.31
Ranson, 2025	TKA	Control (1285269)	64678 (5.0%)	-	-	-	-	-
		GLP-1RA (15387)	687 (4.5%)	-	-	-	-	-
Verhey, 2025	THA	Control (5345)	241 (4.5%)	-	-	310 (5.8%)	-	-
		GLP-1RA (5345)	220 (4.1%)	-	-	255 (4.8%)	-	-

CVA, cerebrovascular event; AKI/ARF, acute kidney injury/acute renal failure; PNA, pneumonia; ED, emergency department.

## Appendix A Supplementary data

Conflict of Interest Statement for All Authors
